# Symmetric Graphene Dielectric Nanowaveguides as Ultra-Compact Photonic Structures

**DOI:** 10.3390/nano11051281

**Published:** 2021-05-13

**Authors:** Da Teng, Yuncheng Wang, Tianzi Xu, Huayu Wang, Qinqin Shao, Yanan Tang

**Affiliations:** College of Physics and Electronic Engineering, Zhengzhou Normal University, Zhengzhou 450044, China; wyc768020@163.com (Y.W); xu991105@gmail.com (T.X); diandianfeihuayu@163.com (H.W.); scu_sqq@163.com (Q.S)

**Keywords:** graphene plasmons, waveguides, subwavelength structures, mid-infrared waves

## Abstract

A symmetric graphene plasmon waveguide (SGPWG) is proposed here to achieve excellent subwavelength waveguiding performance of mid-infrared waves. The modal properties of the fundamental graphene plasmon mode are investigated by use of the finite element method. Due to the naturally rounded tips, the plasmon mode in SGPWG could achieve a normalized mode field area of ~10^−5^ (or less) and a figure of merit over 400 by tuning the key geometric structure parameters and the chemical potential of graphene. In addition, results show that the modal performance of SGPWG seems to improve over its circular counterparts. Besides the modal properties, crosstalk analysis indicates that the proposed waveguide exhibits extremely low crosstalk, even at a separation distance of 64 nm. Due to these excellent characteristics, the proposed waveguide has promising applications in ultra-compact integrated photonic components and other intriguing nanoscale devices.

## 1. Introduction

Plasmonic waveguides (PWGs) [1], which can confine and guide light at the subwavelength scale, are one of the components necessary to realize ultra-high-density photonic integration [2]. The traditional noble metallic PWGs, such as metal stripe/nanowire waveguides [3,4,5], channel/wedge plasmon waveguides [6], gap plasmon waveguides [7,8,9], dielectric-loaded plasmon waveguides [10,11,12], and hybrid PWGs [1,13,14,15,16,17,18], have been intensively studied in the near-infrared and visible bands. However, in the mid- and far-infrared bands, the plasmonic effects of metals (which were modeled as perfect electric conductors) are very weak and the electromagnetic optical response cannot be dynamically adjusted, which imposes restrictions on their applications at the nanoscale [19,20]. Thus, finding appropriate materials for PWGs is an urgent need.

To address this critical challenge, researchers have suggested some available materials with tunable properties for exciting surface plasmons [21], including graphene [22,23,24], transition metal dichalcogenides [25,26,27], bulk Dirac semimetals [28,29], borophene [30,31], etc. Among these, graphene plasmons (GPs) have attracted widespread attention due to their advantages, including strong light–matter interactions, deep subwavelength field confinement, and tunable optical properties [23,24]. Benefiting from these excellent characteristics, graphene has served as an effective nanoscale waveguiding platform in the infrared region. More importantly, the combination of graphene and silicon-on-insulator (SOI) waveguides make it possible to design graphene-based photonic integration devices [32], such as waveguides [33,34,35,36,37,38,39], sensors [40,41], filters [42], modulators [43,44], etc. Recently, graphene–SiO_2_–Si coaxial-like waveguides [45], graphene layer–SiO_2_–Si planar structures [46,47,48,49,50], and graphene-coated nanowires integrated with SiO_2_ or Si substrates [51,52,53,54,55,56] were presented to demonstrate ultra-compact photonic integrated circuits in the mid- and far-infrared bands.

The combination of graphene and silicon-based waveguides not only provides additional degrees of freedom to tune the modal properties, but also leads to strong coupling between the graphene layer and silicon layer, which massively reduces the modal field area. To realize ultra-compact photonic integration, the modal field area should be small enough to circumvent the interference between neighboring structures. Therefore, highly concentrated modal fields are preferred. Here, we propose a symmetric graphene plasmon waveguide (SGPWG) to achieve nanoscale waveguiding of mid-infrared waves. The results of our simulations show that the elliptical nanowire-based SGPWG performs better than its circular counterparts and is suitable for ultra-compact photonics integration. We first introduce the waveguide structure and methods, then comprehensively evaluate the proposed waveguide in the light of geometric and physical parameters. We also discuss the dependences of modal properties on the ratio of the semi-major axis to the semi-minor axis. Finally, we present the crosstalk analysis and briefly compare the mode characteristics of the elliptical nanowire-based SGPWG with those of the circular nanowire-based SGPWG.

## 2. Waveguide Structure and Methods

**Figure 1:**
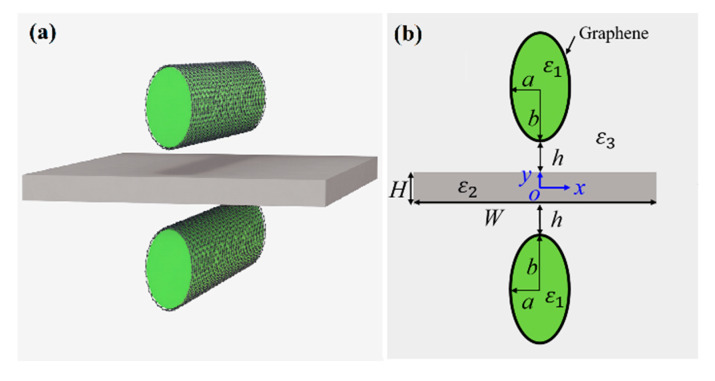
Schematic of the proposed SGPWG. (**a**) Three-dimensional (3D) view, (**b**) two-dimensional (2D) cross-section.

Figure 1 shows the schematic of the proposed SGPWG, which is composed of two graphene-coated elliptical nanowires symmetrically placed on each side of a thin Si slab with a small gap distance, *h*. The semi-minor axis and semi-major axis of the elliptical nanowire are *a* and *b*, respectively. The thickness and width of the Si slab are *H* and *W*, respectively. The relative permittivities of the dielectric nanowire, Si slab, and surrounding silica are set as *ε*_1_ = 2, *ε*_2_ = 12.25 (with an approximated refractive index of 3.5), and *ε*_3_ = 2.25, respectively [57,58,59]. The dielectric nanowires are coated by the monolayer graphene. In this work, the monolayer graphene is simulated as an electric field-induced surface current (**J** = *σ*_g_**E**) on the nanowire surfaces [60]. Graphene’s surface conductivity (*σ*_g_) is obtained from Kubo’s formula, which consists of the intra- and inter-band contributions [56], namely *σ*_g_ = *σ*_intra_ + *σ*_inter_, where:

(1) $$\sigma_{\mathrm{intra}}=\frac{2ie^{2}k_{B}T}{\pi \hbar^{2}(\omega +i/\tau )}\ln[2\cosh(\frac{E_{F}}{2k_{B}T})]$$

(2) $$\sigma_{\mathrm{inter}}=\frac{e^{2}}{4\hbar^{}}[\frac{1}{2}+\frac{1}{\pi }\arctan(\frac{\hbar \omega -2E_{F}}{2k_{B}T})-\frac{i}{2\pi }\ln\frac{{\left(\hbar \omega +2E_{F}\right)}^{2}}{{\left(\hbar \omega -2E_{F}\right)}^{2}+{\left(2k_{B}T\right)}^{2}}]$$

The electron relaxation time *τ* is set at 0.5 ps according to [61]. The temperature is *T =* 300 K; *ω =* 2π*f,* with *f* being the frequency of the incident light; *E*_F_ is the chemical potential; *ℏ* is the reduced Plank’s constant; *k*_B_ is the Boltzmann’s constant; and *e* = 1.6 × 10^−19^ C.

The GP mode propagates along the *z*-direction with a complex propagation constant *k*_z_, where: *k*_z_ = *k*_0_*N*_eff_; *k*_0_ = 2π/*λ*_0_, where *λ*_0_ is the wavelength in air; and *N*_eff_ is the complex effective mode index. The propagation distance is calculated by *L*_P_ = *λ*_0_/[2πIm(*N*_eff_)], where Im(*N*_eff_) denotes the imaginary part of *N*_eff_. The normalized mode size is defined as *A*_N_ = *A*_eff_/*A*_0_, where *A*_0_ = *λ*_0_^2^/4, and *A*_eff_ is defined as

(3) $$A_{\mathrm{eff}}=\iint W(r)d^{2}r/\max\{W(r)\}$$

where *W*(r) represents the energy density of the plasmon mode [1]. Figure of merit (FoM) [62] is defined as *L*_P_/(*A*_eff_/π)^1/2^. The modal properties are investigated by the finite element method (FEM) software COMSOL Multiphysics, which is capable of precisely modeling complex plasmonic structures and thus widely used [1,60,63].

## 3. Results and Discussion

**Figure 2:**
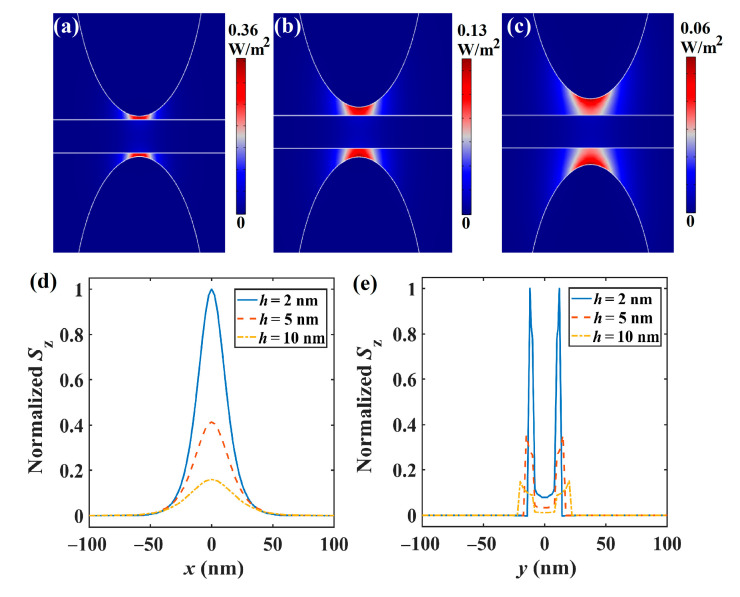
Energy density distribution. (**a**–**c**) Two-dimensional energy density distributions when *h* = 2, 5, and 10 nm. (**d**–**e**) Normalized energy density distributions along the *x* (at *y* = 0 nm) and *y* (at *x* = 0 nm) directions (shown in Figure 1b).

The time-averaged Poynting vector along the *z*-direction is given as *S*_z_ = 1/2Re(**E** × M*)|_z_, where Re(·) is the real part, the superscript of **M** is the complex conjugate, and **E** and **M** are the electric and magnetic field vectors. Figure 2a–c depicts the 2D energy density (*S*_z_) distributions of the fundamental mode in SGPWG for different gap distances (*h* = 2, 5, 10 nm), where *a* = 50 nm, *b* = 100 nm, *W* = 400 nm, *H* = 20 nm, *E_F_* = 0.5 eV, and *f* = 30 THz. It could be seen that the optical energy of the plasmon mode is mainly concentrated between the nanowire and the Si layer. For *h =* 2, 5, and 10 nm, the peak values of the energy density are 0.36, 0.13 and 0.06 W/m^2^, respectively. To get an intuitive view, Figure 2d,e depicts the normalized energy density distributions along the *x* (at *y* = 0 nm) and *y* (at *x* = 0 nm) directions (shown in Figure 1b), respectively. Clearly, the energy is mainly restricted between the nanowires. With increasing *h*, the field confinement weakens, and the energy density becomes more dispersed. Both features are consistent with the coupling behavior between metal nanoparticles [64]. This is because the coupling strength between the GP mode and silicon layer decreases when *h* increases. In order to increase the degree of field confinement, the gap should be at the deep subwavelength scale (e.g., 2~20 nm), which in turn can help to avoid crosstalk between optical signals in the photonic integrated circuits.

As elliptical nanowires are involved in the proposed SGPWG, we need to consider different *b*/*a* values. We chose four representative *b*/*a* values throughout the paper, namely *b*/*a* = 0.5, 1, 2, and 5. Although the case of *b*/*a* = 1 has already been investigated in [52], we show that the *b*/*a* > 1 setting presented an increased subwavelength optical energy transmission performance as compared to the *b*/*a* = 1 setting.

**Figure 3:**
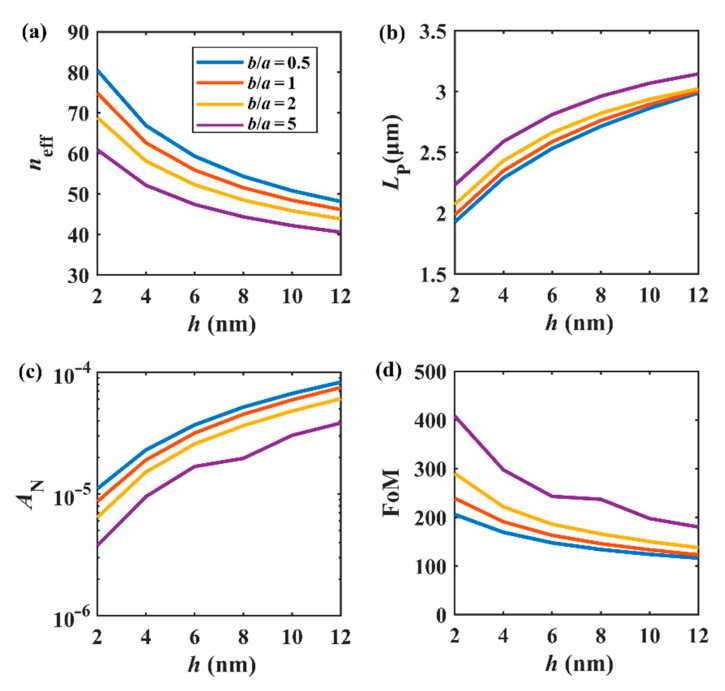
Modal properties of *h* at different *b*/*a* values. (**a**) *n*_eff_, (**b**) *L*_P_, (**c**) *A*_N_, (**d**) FoM.

Figure 3 shows the relationship between the fundamental mode properties and the gap distance *h* at different *b*/*a* values (*b*/*a* = 0.5, 1, 2, and 5). The parameters were *a* = 50 nm, *W* = 400 nm, *H* = 20 nm, *E*_F_ = 0.5 eV, and *f* = 30 THz. From Figure 3a, it can be seen that the real parts of the effective mode indices (*n*_eff_) decrease with the increase in *h* for *b*/*a* = 0.5, 1, 2, and 5. For the case of *b*/*a* = 0.5, the coupling between the plasmon mode and the Si layer is much stronger, and then gradually weakens with the increase in *b*/*a*. For the four cases considered here, *L*_P_ increases with the increase in *h*. As a whole, the propagation distance also increases with the increase in *b*/*a*, as shown in Figure 3b. Figure 3c,d shows the normalized mode area and figure of merit, respectively. As stated above, the smaller the gap distance, the better the field confinement. Therefore, one can see that *A*_N_ varies from 3.76 × 10^−6^ to 8.305 × 10^−5^ when *h* increases from 2 to 12 nm. At a fixed *h* value, the normalized mode area decreases with the increase in *b*/*a*. Finally, the plots of figure of merit shown in Figure 3d indicate that the overall performance of the SGPWG can be improved when *h* is reduced.

Based on above simulations, we show that the cases of *b*/*a* > 1 (orange and purple lines) show better performances compared with the cases of *b*/*a* = 1 (red lines) and 0.5 (blue lines) in terms of *L*_P_, *A*_N_, and FoM. In other words, these results show that the modal performance of elliptical nanowire-based SGPWG is better than its circular counterparts. Noticing that the FoM decreases with the increase in the gap distance *h*, we set *h* = 5 nm in the following experiments to maintain the good performance of this structure.

**Figure 4:**
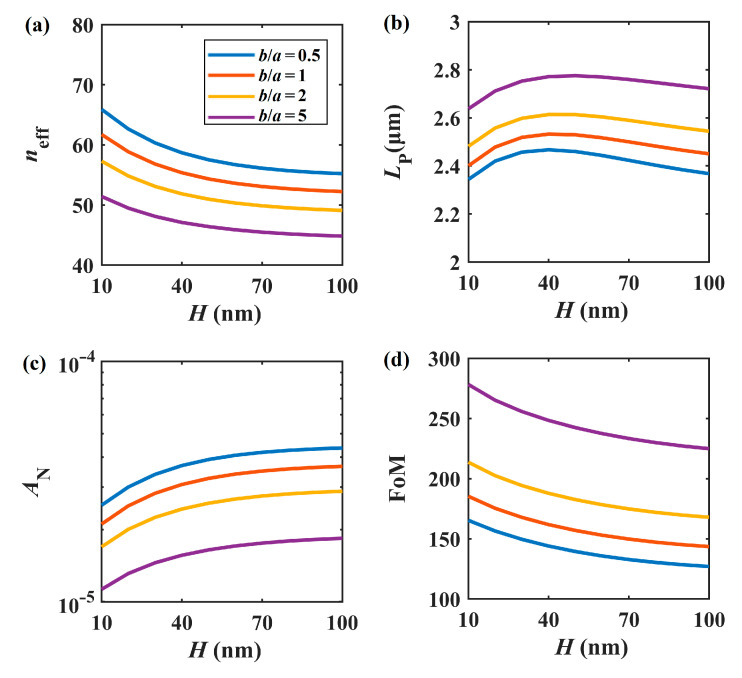
Modal properties of *H* at different *b*/*a* values. (**a**) *n*_eff_, (**b**) *L*_P_, (**c**) *A*_N_, (**d**) FoM.

Figure 4 shows the relationship between the fundamental mode properties and the thickness of the silicon layer at *b*/*a* = 0.5, 1, 2, and 5 when *a* = 50 nm, *W* = 400 nm, *h* = 5 nm, *E*_F_ = 0.5 eV, and *f* = 30 THz. The modal properties are similar to those presented in Figure 3, except for Figure 4b, where the propagation length seems to have a maximum value when *H* is around 30 nm. As show in Figure 4b,c, when *H* increases, *L*_P_ first reaches a maximum value and then slightly decreases, while the normalized mode area *A*_N_ increases monotonically. With increasing *H*, the corresponding effective mode index and FoM decrease significantly, as shown in Figure 4a,d. Hence, we set *H* = 20 nm to maintain a large FoM value in the following experiments. Apparently, *b*/*a* > 1 settings (orange and purple lines) perform better than *b*/*a* = 1 (red lines) and 0.5 (blue lines) settings in terms of *L*_P_, *A*_N_, and FoM.

**Figure 5:**
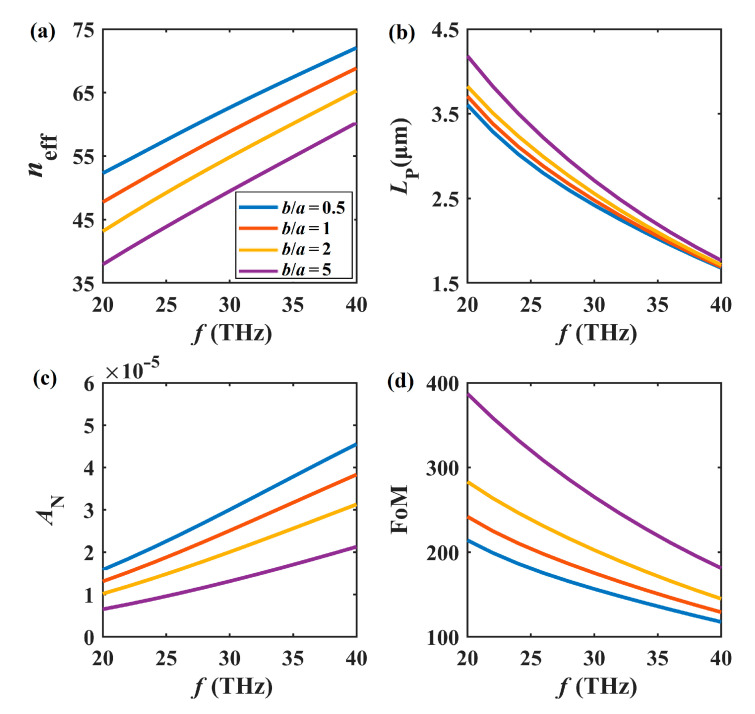
Modal properties of *f* at different *b*/*a* values. (**a**) *n*_eff_, (**b**) *L*_P_, (**c**) *A*_N_, (**d**) FoM.

The frequency-dependent mode characteristics of the SGPWG are shown in Figure 5 at different *b*/*a* values. We set *a* = 50 nm, *W* = 400 nm, *H* = 20 nm, *h* = 5 nm, and *E*_F_ = 0.5 eV. As shown in Figure 5a,b, when the frequency varies from 20 to 40 THz, *n*_eff_ increases linearly and *L*_P_ decreases monotonically. This is because at higher frequencies, the large absorption of graphene leads to the increase in propagation loss. From Figure 5c, we see that the normalized mode field area (*A*_N_) increases with increasing frequency, maintaining a level of ~10^−5^. As for the figure of merit shown in Figure 5d, increasing frequency degenerates the overall performance. In other words, higher frequencies lead to higher *n*_eff_ and *A*_N_ values, but lower *L*_P_ and FoM values. Once again, we showed that *b*/*a* > 1 settings (orange and purple lines) perform better than *b*/*a* = 1 (red lines) and 0.5 (blue lines) settings in terms of *L*_P_, *A*_N_, and FoM.

**Figure 6:**
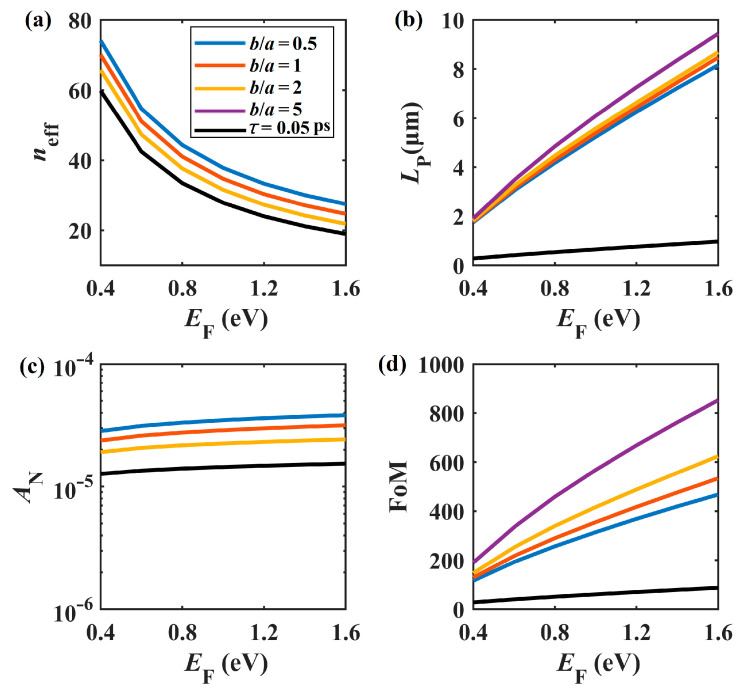
Modal properties of *E_F_* at different *b*/*a* values. (**a**) *n*_eff_, (**b**) *L*_P_, (**c**) *A*_N_, (**d**) FoM. Note that the black lines overlap the purple lines in (**a**) and (**c**).

To present the tunability of the GP mode in the proposed SGPWG, the mode properties versus *E*_F_ are presented in Figure 6. In [65], the chemical potential of graphene could reach a value of 1.77 eV; thus, *E*_F_ varied from 0.4 to 1.6 eV. The other parameters were *a* = 50 nm, *W* = 400 nm, *H* = 20 nm, *h* = 5 nm, and *f* = 30 THz. As shown in Figure 6a,b, when *E*_F_ changes from 0.4 to 1.6 eV, both *n*_eff_ and modal loss decrease monotonically. The latter is due to the fact that when the chemical potential is increased, the interband contribution of *σ*_g_ is drastically reduced, thus leading to the reduction of propagation loss. However, the chemical potential seems to have a limited effect on *A*_N_ (see Figure 6c), since the modal field area changes a little. In addition, the figure of merit shown in Figure 6d increases rapidly with *E*_F_ increase, and reaches a value of 850 when *E*_F_ = 1.6 eV and *b*/*a* = 5. Overall, when *b*/*a* ranges from 0.5 to 5, *L*_P_ and FoM increase and *A*_N_ decreases, indicating that the elliptical nanowire-based SGPWG performs better than its circular counterparts. A recent report showed that the graphene samples fabricated by large scale methods show a relaxation time of only *τ* = 0.05 ps [66]. Hence, we also studied the modal properties when *τ* = 0.05 ps (see black lines) and *b*/*a* = 5 for comparison. As seen in Figure 6b, *L*_P_ substantially decreases while *n*_eff_ and *A*_N_ are nearly unchanged (see Figure 6a,c), and the black lines are overlapped with the purple lines. These results indicate that the shorter relaxation time degenerates the overall performance of the SGPWG (see Figure 6d). This is because the imaginary part of the equivalent relative permittivity of monolayer graphene increases about ten times.

**Figure 7:**
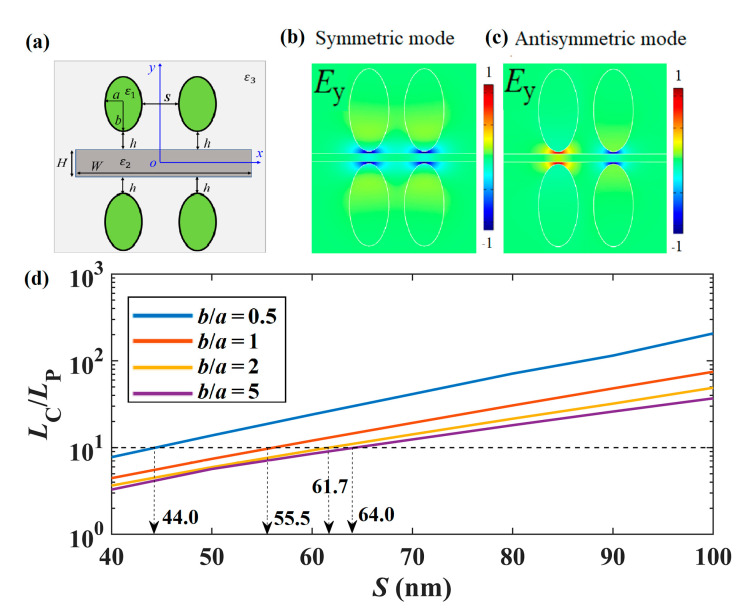
Crosstalk between SGPWGs. (**a**) Coupling system; (**b**) and (**c**) for normalized *E*_y_ field distributions of symmetric and antisymmetric modes when *b*/*a* = 2 and *S* = 40 nm; (**d**) *L*_C_/*L*_P_ with respect to *S* for different *b*/*a* values.

If ultra-compact photonic integration is attempted, highly localized modal fields could help reduce the crosstalk between neighboring structures. Here, we studied the crosstalk between neighboring structures by considering a system consisting of two parallel SGPWGs with an edge-to-edge distance of *S* (40~100 nm) as shown in Figure 7a. The parameters were *a* = 50 nm, *W* = 800 nm, *H* = 20 nm, *h* = 5 nm, *E*_F_ = 0.5 eV, and *f* = 30 THz. The electric field (*E*_y_) distributions of the symmetric and antisymmetric modes are depicted in Figure 7b,c with *S* = 40 nm and *b*/*a* = 2. Based on the coupled mode theory, the crosstalk is estimated by the coupling length *L*_C_, which is the length required for complete power transfer from one waveguide to the other, and is given by *L*_C_ = *λ*_0_/(2|*n*_eff,s_ - *n*_eff,as_|), where *n*_eff,s_ and *n*_eff,as_ denote the real parts of the effective mode indices of the symmetric and antisymmetric modes, respectively [7]. Figure 7d shows the normalized coupling lengths (*L*_C_/*L*_P_) with respect to *S* for different *b*/*a* values. Usually, when *L*_C_/*L*_P_ approaches 10 (see the black dashed line), it is assumed that no coupling happens between the neighboring components. When *L*_C_/*L*_P_ reaches 10, the corresponding edge-to-edge distance are about 44.0, 55.5, 61.7, and 64.0 nm for *b*/*a* = 0.5, 1, 2, and 5, respectively. It is worth noting that when *S* is above 64 nm, *L*_C_/*L*_P_ is always larger than 10, which indicates that the proposed SGPWG shows extremely low crosstalk between neighboring components and is suitable for ultra-compact photonics integration.

**Figure 8:**
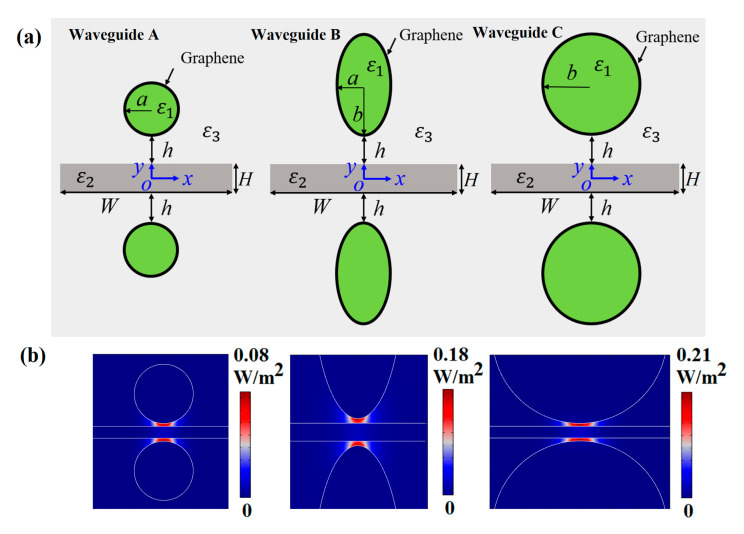
Comparison of three SGPWGs. (**a**) Cross-section of Waveguide A (*R* = *a* = 50 nm), B (*a* = 50 nm, *b* = 150 nm), and C (*R* = *b* = 150 nm); (**b**) 2D energy density distributions of (**a**).

Next, we briefly compare the mode characteristics among three different kinds of waveguides, including circular nanowire-based SGPWGs with *R* = *a* (Waveguide A) and *R = b* (Waveguide C), and an elliptical nanowire-based SGPWG (Waveguide B). The schematics of the three SGPWGs are shown in Figure 8a, and we set *a* = 50 nm, *b* = 150 nm, *W* = 400 nm, *h* = 5 nm, *H* = 20 nm, *E*_F_ = 0.5 eV, and *f* = 30 THz. Figure 8b shows the 2D energy density distributions of the fundamental mode in three SGPWGs. The results show that Waveguide B exhibits much stronger energy confinement than Waveguides A and C, which can be intuitively seen from Table 1 (lower *A*_N_). As shown in Table 1, the plasmon mode in Waveguide B has the smallest normalized mode area and largest *L*_P_ compared with the other waveguides.

Finally, we briefly introduce the fabrication process of the SGPWG. Recent reports [67,68] showed that graphene-coated elliptical nanowires can be experimentally made by coating a dielectric nanowire with a monolayer of graphene due to van der Waals forces. Then, based on modern semiconductor fabrication technology, the substrate silica can be deposited on top of the buffer layer [69]. After that, one GCNW is placed on the silica substrate, then the GCNW is covered by silica deposition with a thickness of 2*b* + *h*. Next, a silicon layer with a thickness of *H* (about 10 nm) could be deposited on top of the silica using plasma-enhanced chemical vapor deposition (PECVD) technology [70]. Then, *h*-thick silica is deposited on the silicon layer before transferring another GCNW. Finally, the second GCNW is covered by silica deposition with a thickness of over 2*b*.

## 4. Conclusions

In summary, a symmetric graphene plasmon waveguide is proposed and investigated. The simulation results show that a normalized mode field area of ∼10^-^^5^ and a figure of merit of ~400 can be achieved by optimizing the parameters. The mode characteristics could be dynamically tuned by changing the chemical potential of graphene by a DC bias voltage or chemical doping. By varying the ratio of *b*/*a*, the simulation results show that elliptical nanowire-based SGPWGs (*b*/*a >* 1) show better performance compared with the cases of *b*/*a* ≤1 in terms of *L*_P_, *A*_N_, and FoM. In addition, crosstalk analysis suggests that the proposed SGPWGs have extremely small energy couplings between neighboring components, even at a separation distance of 64 nm. These findings could have potential applications for ultra-compact photonic integration and subwavelength optoelectronic devices in the mid-infrared band.

## Figures and Tables

**Table 1:** Waveguiding performance comparison of three SGPWGs.

Waveguide	*N*_eff_	*L*_P_/μm	*A*_N_
A	58.845 + 0.642*i*	2.477	2.512 × 10^−5^
B	52.428 + 0.608*i*	2.616	1.699 × 10^−5^
C	65.394 + 0.658*i*	2.417	3.501 × 10^−5^

## Data Availability

The data presented in this study are available on request from the corresponding author.
